# Antioxidant, Anti-Obesity, and Hypolipidemic Effects of Polyphenol Rich Star Anise (*Illicium verum*) Tea in High-Fat-Sugar Diet-Induced Obesity Rat Model

**DOI:** 10.3390/antiox11112240

**Published:** 2022-11-14

**Authors:** Neelam Iftikhar, Abdullah Ijaz Hussain, Ghulam Mustafa Kamal, Sidra Manzoor, Tabinda Fatima, Farhan Khashim Alswailmi, Ashfaq Ahmad, Bader Alsuwayt, Sulaiman Mohammed Abdullah Alnasser

**Affiliations:** 1 Natural Product and Synthetic Chemistry (NPSC) Lab, Department of Chemistry, Government College University Faisalabad, Faisalabad 38000, Pakistan; 2 Central Hi-Tech Lab, Government College University Faisalabad, Faisalabad 38000, Pakistan; 3 Institute of Chemistry, Khwaja Fareed University of Engineering and Information Technology, Rahim Yar Khan 64200, Pakistan; 4 Department of Pharmaceutical Chemistry, College of Pharmacy, University of Hafr Al-Batin, Hafr Al-Batin 39524, Saudi Arabia; 5 Department of Pharmacy Practice, College of Pharmacy, University of Hafr Al-Batin, Hafr Al-Batin 39524, Saudi Arabia; 6 Department of Pharmacology and Toxicology, Unaizah College of Pharmacy, Qassim University, Buraidah 51452, Saudi Arabia

**Keywords:** BMI, VLDL, HDL, kidney index, liver index, high-fat diet, DPPH radical scavenging activity, phenolic acids and flavonoids

## Abstract

Star anise (*Illicium verum* Hook. fil.) is commonly utilized as a culinary and medicinal fruit and is most famous in indigenous systems of medicine. The present research work aims to appraise and validate the potential of polyphenol-rich star anise tea (SAT) on oxidative stress, obesity and related biochemical parameters in high-fat-sugar-diet (HFSD)-induced obesity model in rats. SAT was prepared using the traditional method in warm water. The Reverse Phase High Pressure Liquid Chromatography (RP-HPLC) analysis was performed for the simultaneous determination of phenolic acids and flavonoids in SAT. Two doses (250 and 500 mg/kg body weight) were selected to investigate the anti-obesity potential of SAT using HFSD-induced obese rat model. Major (>5 mg/100 mL) phenolic acids in SAT were *p*-coumeric acid, gallic aid, cinamic acid, chlorogenic acid and ferulic acid while catechin and rutin were the major flavonoids detected in the SAT. SAT exhibited 51.3% DPPH radical scavenging activity. In vivo study showed that higher doses of SAT (500 mg/kg body weight) significantly reduced the body weight increase (74.82%) and BMI (0.64 g/cm^2^). Moreover, significant reductions in the levels of serum total cholesterol, triglyceride, LDL and VLDL were recorded in all the treatment groups in comparison to the HFSDC group. Furthermore, SAT reduced the alterations in MDA, SOD and GSH levels of experimental groups thus showing the potential against oxidative stress. The SAT-500 group showed a significant decrease in the elevated kidney and liver weights and atherogenic index in comparison to the HFSDC group. The present study proved that SAT exhibited strong protective effects against obesity and oxidative stress, especially at higher doses.

## 1. Introduction

Obesity is a complex and chronic disorder influenced by behavioral, genetic and environmental factors [1]. It is one of the burning issues of public health problems of the running century, affecting all age groups and correlated with oxidative stress that further results in other complications in the form of chronic heart diseases, certain types of cancers (estimated 41% cases), type 2 diabetes (estimated 44% cases), obstructive sleep apnea, osteoarthritis, and psychiatric diseases [2,3,4,5]. Moreover, obesity is an evolving risk factor for vulnerability and the severity of coronavirus disease 2019 (COVID-19) caused by the acute respiratory syndrome coronavirus-2 (SARS-CoV-2) and it generates alterations in the microbiota and immune responses that are linked with poor virus responses [6,7,8]. Obesity and overweight are indiscriminately defined via the weight gain and body mass index (BMI). Body mass index is defined as a person’s weight in kilograms (kg) divided by the square of their height in meters (kg/m^2^). BMIs of 25, 30 and 40 kg/m^2^ were generally classified as overweight, obesity and morbid obesity (currently type III obesity), respectively [1]. 

Different practices are in use to control obesity-related issues, comprising several types of surgeries, hard workouts and utilization of natural products and medications (limonabant, sibutramine and orlistat) [9,10]. Nevertheless, there are some obstacles in the implementation of some of these methodologies because of a lack of awareness, the easy and busy life routine of individuals, high cost of conventional drugs and their side effects [9,11]. Currently, the only medication authorized by the European Medicines Agency (EMA) for the treatment of chronic obesity is orlistat but it has also been associated with some side effects such as gastrointestinal reactions, oily spotting, abdominal cramps and liquid stools [12,13]. Moreover, its pharmacological effects rely on patient compliance with certain dietary restrictions, limiting its efficacy [12]. Hence, plant-based medications and natural products have again attracted the attention of natural product scientists and doctors as a safe therapeutic strategy for the management of several diseases including obesity [14]. 

Polyphenols are secondary metabolites that carry one or more hydroxyl groups and are common in the plant kingdom. Polyphenols are further classified into two main groups, that is, flavonoids (e.g., flavanols, flavanols, flavanones, flavanones, isoflavones and anthocyanins) and non-flavonoids (e.g., phenolic acids, stillbenes, tannins, lignans, and xanthones). The use of plants’ bioactive constituents including polyphenols is gaining more interest day by day due to their prescribed role in human health research [1,5,15,16]. The relationship between polyphenols consumption and human health has been reported with special reference to oxidative stress, hypertension, cardiovascular diseases, diabetes, cancers and obesity [1,5,16,17,18]. In obesity, flavonoids and phenolic acids could regulate adipocyte metabolism to limit the growth of adipose tissue and are being used in the improvement of various natural weight management products [16,17]. Therefore, polyphenols or polyphenol-rich products can be safe, affordable, efficient and economical anti-obesity agents [14]. 

*Illicium verum* Hook. fil. (Commonly named star anise), belongs to the genus Illicium of the *Illiciaceae* family, is an evergreen tree having red-purple flowers and anise-scented star-shaped fruit which is native to the southwest of China and Vietnam [19,20]. It has been widely used spice throughout the world and in traditional medicines for the treatment of stomach aches, carminative, dyspepsia, insomnia, sleeplessness, skin inflammation, antirheumatic, and diuretic [21,22,23,24,25]. It was also tested to have antifungal, antibacterial, antiseptic, chemopreventive, anti-flu, anticoagulant, insecticidal and anti-HIV (human immunodeficiency virus) activities [24,26]. Phytochemical analysis showed that star anise is a rich source of polyphenols, and terpenoids like trans-anethole that possess various biological activities including antithrombotic, antihypertensive, antihyperlipidemic and anti-obesity activities [23,27,28]. 

To the best of our information, no report is presented on the anti-obesity effect of the star anise and its polyphenols. Therefore, the present research work was planned to investigate the phenolic profile, DPPH free radical scavenging capacity and anti-obesity activity of star anise tea using a high-fat-sugar diet-induced obesity model in WKY rats. Moreover, complete biochemical and histopathological analyses were also performed to confirm the effect of star anise polyphenols.

## 2. Materials and Methods

### 2.1. Collection and Identification of Plant Materials

Fruits of star anise (*Illicium verum*) were collected from the Botanical Garden of Government College University, Faisalabad, Pakistan. The authenticated sample was secured in sealed polythene bags and moved to the Natural Products Research Laboratory (NPSC), GC University, Faisalabad, Pakistan. 

### 2.2. Reagents, Reference Compounds and Chemicals

Standards and reference chemicals used in this study, for example, quercetin, catechin, myricetin, kaempferol, ferulic acid, *p*-hydroxy benzoic acid, gallic acid, sinapic acid, chlorogenic acid, *p*-coumeric acid, vanillic acid, caffeic acid, ascorbic acid, linoleic acid (60–74%), orlistat, 2,2-diphenyl-1-picrylhydrazyl radical (DPPH•), Folin-Ciocalteu reagent, butylated hydroxytoluene (BHT), Tween 80 were acquired from Sigma Chemical Co. (St Louis, MO, USA). All other chemicals were of analytical grades and purchased from Merck (Darmstadt, Germany), and used without further purification.

### 2.3. Preparation of Star Anise Tea

Star anise tea (SAT) was prepared as reported previously [16]. Briefly, a 20-g dried and grounded plant sample (mesh size 80) was shaken in 200 mL distilled water in an orbital shaker (Gallenkamp, UK) for continuous agitation at 180 rpm for 28 h at 55 °C temperature. After filtering the solid residues, the solutions were dried using a rotary evaporator (Eyela, SB-651, Rikakikai Co. Ltd., Tokyo, Japan) under reduced pressure and temperature to get a dried extract. The yield of extract was calculated on the dry plant material basis using the following formula;

Yield (g/100g) = (Weight of dry extract)/(Weight of dry plant material) × 100

### 2.4. Simultaneous HPLC ANALYSIS of Phenolic Acids and Flavonoids

The hydrolysis of star anise extract was carried out as reported previously [15,29]. The extract solution (10 mg/mL) was filtered through a 0.45 µm non-pyrogenic filter (Minisart, Satorius Stedim Biotech GmbH, Goettingen, Germany). The HPLC analysis was performed with Flexar Perkin Elmer System (Perkin Elmer, Shelton, CT, USA) equipped with gradient model Flexar pumps system, LC-Shelton CT, 06484 (USA) UV/Visible detector, column oven and degasser (DG-20A5) systems. A hypersil GOLD C18 column (250 × 4.6 mm internal diameter, 5 µm particle size) (Thermo Fisher Scientific Inc., Waltham, MA, USA) 20 µL of the filtered extract solution was injected into the injection loop, and a non-linear gradient containing acetonitrile::methanol (70:30) and water with 0.5% glacial acetic acid was used as mobile phase. UV spectra were recorded at 275 nm. Stock solutions of all the standards were freshly prepared by dissolving reference compounds in methanol (1 mg/mL). Working standard solutions were made by gradual dilution with methanol to the required concentration between 0.4 to 100 µg/mL and the calibration curve of each standard was formed by plotting the concentration of the standard against the peak area. Two techniques were employed to identify the compounds, that is, spiking the samples with standards and matching the retention times with the reference compounds whereas, quantification was done using the standard curves of reference compounds by the standard addition method.

### 2.5. In Vitro Antioxidant Activity

#### 2.5.1. Determination of Total Phenolic and Flavonoid Contents

The total phenolic (TP) and total flavonoid (TF) contents of SAT extract were measured using methods as described previously [16]. For TP contents, the standard curve of gallic acid solution (10–80 ppm concentration) and results were calculated using equation (y = 0.026x + 0.000, R^2^ = 0.997) and reported as mg of TP contents per gram of plant material, measured as gallic acid equivalent. Similarly, for TF contents standard curve of catechin solution (10–160 ppm concentration) was prepared and results were calculated using equation (y = 0.006x + 0.015, R^2^ = 0.999) and reported as mg of TF contents per gram of plant material, measured as catechin equivalent. 

#### 2.5.2. DPPH Free Radical Scavenging Capacity

DPPH (2,2-Diphenyl-1-picrylhydrazyl) radical scavenging activity of SAT was performed as reported previously [15]. Two milliliters (mL) of 90 μM DPPH solution in methanol was mixed with SAT extract and BHT solution (10 µg/mL) separately and incubated for ½ h at room temperature. The absorbance was recorded at λ_max_ (517 nm) and scavenging was calculated using the following formula;

Scavenging (%) = (Absorbance of DPPH solution − Absorbance of sample solution)/(Absorbance of DPPH solution) × 100

### 2.6. In Vivo Anti-Obesity Activity

In vivo study on rats was performed in accordance with the guidelines of the Institutional Review Board for Animal Studies (Study No 19680/IRB No 680), Government College University Faisalabad, Pakistan. For preliminary in vivo acute oral toxicity study (sighting study) and to calculate the effective dose, the SAT was administered at doses of 50, 300, 500, and 2000 mg/kg/p.o. as reported and observed the behavioral change, physical appearance, weight loss, hair fall, redness of the eyes and water intake [16,30]. SAT was found to be nontoxic up to the maximum dose of 2000 mg/kg body weight.

#### 2.6.1. Composition of Normal and High Fat Diet for Rats

The composition of standard rat chow was purchased from a store and its composition was vitamin mixture (1%), mineral mixture (4%), cellulose (5%), corn oil (5%), sucrose (9%), casein (26%) and corn starch (50%). The high-fat diet was prepared in pellet form, and its composition was vitamin mixture (1%), mineral mixture (4%), cellulose (5%), sucrose (9%), corn starch (15%), casein (26%) and beef tallow (40%). Food was stored at 24 °C in sealed containers. To increase the sugar contents in the diet, the rats were also administrated a carbonated soft drink (coke) solution (50:50 *v*/*v*) in water. Thus, the high-fat diet contained more lipids with an energy difference of 4.37 KJ/g in comparison to the normal diet and can be categorized as a hyper-caloric diet. 

#### 2.6.2. Animals and Experimental Design

Adult, three weeks old (130–160 g) male Wistar Kyoto (WKY) rats were acquired from Animals House, University of Veterinary and Animal Sciences, Lahore (UVAAS). Animals were kept in 41 × 34 × 16 cm polypropylene cages (six rats in each cage) under constant temperature (25 ± 2 °C) and humidity (65 ± 5%) and rat chow was freely available to all the rats with water ad libitum. Rats were acclimatized for one week in the animal transit room and were then divided randomly into five groups and six rats were selected in each group. All the rat groups were provided soft drink: water (1:1) solution ad libitum throughout the study period except the control group, which was only provided water ad libitum. The SAT and orlistat were given to the respective treatment group through oral gavage.

Normal Control (NC) group [Received normal feed (approx. 20 g/rat/day)].

High-fat diet Control (HFDC) group [Received HFD (approx. 20 g/rat/day)].

Positive Control (PC) group [Received HFD (approx. 20 g/rat/day) plus orlistat 250 mg/kg Body Weight/day for 28 days].

SA-250 group [Received HFD (approx. 20 g/rat/day) supplemented with star anise extract (250 mg/kg BW/day for 28 days)].

SA-500 group [Received HFD (approx. 20 g/rat/day) supplemented with star anise extract (500 mg/kg BW/day for 28 days)].

### 2.7. Observations Recorded 

#### 2.7.1. Obesity Parameters

The percentages of body weight gain and body mass index (BMI) were measured as indicators of obesity as reported previously [16]. The individual body weight of each rat was recorded on days 1, 7, 14, 21 and 28 and the average weight gain of each group was calculated.

Weight gain (%) = (Weight at day 28 (g) − Weight at day 1 (g))/(Weight at day 1 (g)) × 100

Body mass index (BMI) was measured at the end of experiment as reported [16].

#### 2.7.2. Collection of Blood and Tissues Samples

After 28 days, the rats were fasted overnight however water was available to them freely. Blood samples were collected from the right carotid artery, under anesthesia and the blood was centrifuged for 15 min at 3000 rpm. A clear layer of serum was collected into tubes and stored at −70 °C for further analysis. The liver and kidneys were rapidly dissected, washed with normal saline, and cleared from connective tissues and blood clots before weight. The tissues were stored in 10% formalin until histological examination was performed. The animals were then euthanized by exsanguinations under anesthesia. 

The kidney index (KI) and liver index (LI) were calculated using the following formulas.

KI (%) = (Average kidney weight (g))/(Rat weight (g)) × 100

LI (%) = (Liver weight (g))/(Rat weight (g)) × 100

### 2.8. Biochemical Investigations

#### 2.8.1. Estimation of Cholesterol 

Serum was used for the estimation of the following biochemical parameters using a semi-auto analyzer [16]. Total cholesterol (TC) and triglyceride (TGL) were estimated by the cholesterol esterase method and glycerol-3-phosphate Oxidase method, respectively. High-density Lipoprotein (HDL) cholesterol was estimated by using diagnostic kits based on the phosphortungstate method (Bayer Diagnostics Ltd., Karachi, Pakistan), whereas, Friedewald’s formulae were used for the estimation of low-density Lipoprotein (LDL) and Very Low-Density Lipoprotein (VLDL) cholesterols, as given below [31].

LDL Cholestrol (mg/dl) = Total serum cholestrol − HDL cholestrol − (Total serum triglyceridesl)/5

VLDL Cholestrol (mg/dl) = (Total serum triglycerides)/5

#### 2.8.2. Estimation of Oxidative Stress Parameters 

The oxidative status of the rats was determined as reported previously [14]. Measurement of lipid peroxidation product, malondialdehyde (MDA) was done for evaluating the oxidative damage of lipids [16]. Both enzymatic and non-enzymatic defense against oxidative stress were estimated by measuring superoxide dismutase (SOD) and reduced glutathione (GSH) levels using reported protocols [16]. The method of Miller et al. [32] was used to determine the total antioxidant capacity (TAC).

#### 2.8.3. Estimation of Liver and Kidney Functions

Serum creatinine and alkaline phosphatase levels were checked to access the function of the kidneys. To evaluate the function of the liver, serum alanine aminotransferase (ALT), aspartate aminotransferase (AST) and bilirubin total (BT) levels were estimated as reported previously [16].

### 2.9. Histopathology of Liver and Kidney Tissues

Kidney and liver tissues were removed from formalin solution, washed in running tap water, dehydrated in serial dilutions of ethyl alcohol and cleaned with Xylene as reported previously [16]. Mayer’s egg albumin was used for mounting the tissue sections on labeled glass slides and then tissues were cut into thin sections (5–15 µm thickness) using a microtome. Finally, for morphological investigation, stained slides were observed under a light microscope.

### 2.10. Statistical Analysis

Three samples of the plant material were collected and processed individually in triplicate. The in vitro trials were also conducted in triplicates and the data are reported as mean value with standard deviation. One way and two ways Analysis of Variance (ANOVA) followed by Bonferroni/Dunnett (all mean) post hoc test using STATISTICA 5.5 (Stat Sift Inc, Tulsa, OK, USA) were applied, and the differences between the means were considered statistically significant at probability value *p* ≤ 0.05. Linear regression analysis and analysis of covariance (ANCOVA) were performed by using SPSS-16.

## 3. Results and Discussion

### 3.1. Aqueous Extract Yield and Antioxidant Activity

The extract yield (g/100g) of star anise was 4.83 g/100 g of dry plant material (Table 1). Chung (2009) [33] reported a 16.4% water extract yield from dried star anise powder. The variation in the yield of extract possibly is due to the difference in the extraction process and the geographical variation of the plants.

The total phenolic (TP) and total flavonoid (TF) content of star anise tea was evaluated by Folin-Ciocalteu and aluminum chloride methods, respectively. The results were presented in Table 1 and reported as mg of TPC/g of dry plant material, measured as gallic acid equivalent (GAE) and mg of TFC/g of dry plant material, measured as catechin equivalent (CE). Aqueous extract of star anise showed 0.83 mg TPC/g of plant material as GAE while 1.24 mg TFC/g of dry plant material, as CE. Phenolic compounds are ubiquitous in plants, and gaining interest due to their antioxidant properties and physiological and morphological importance in plants [34]. So, it is not only beneficial to quantify phenolic contents but also to assess its contribution to antioxidant activity [35]. Chung [32], reported that the TPC in the aqueous extract of star anise was 114.6 mg/g of extract whereas Kanatt [34] reported the phenolic content in the water extract of star anise to be 237.69 mg/g of extract and the flavonoid content was 115.8 mg/g of extract. These variations might have been due to the variation in the geographical, seasonal and agroclimatic conditions of the species.

Free radical scavenging activity of SAT and BHT solution (10 µg/mL) are presented in Table 1. Star anise showed 51.3% radical scavenging activity whereas, synthetic antioxidant BHT showed significantly higher activity, that is, 89.3%. More than 50% scavenging at 10 µg/mL extract concentration is considered a potential antioxidant extract. Chung [33] reported that 1 mg/mL star anise water extract showed 54.36% radical scavenging activity which is lower than the present investigations. 

### 3.2. HPLC Analysis of Phenolic Acids and Flavonoids

**Figure 1:**
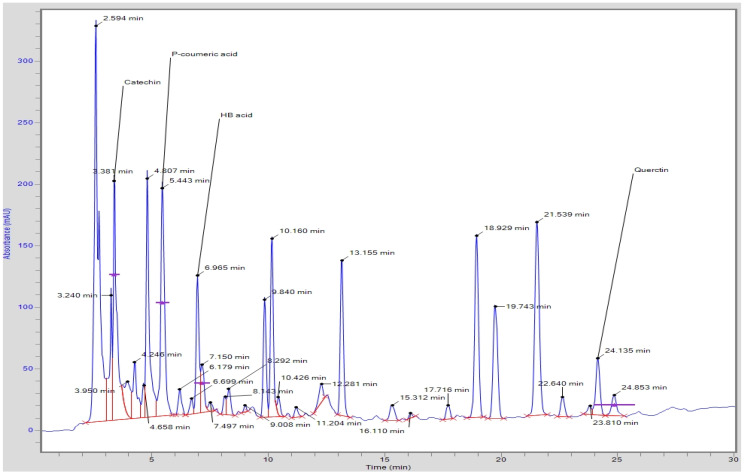
Typical HPLC chromatogram showing the separation of polyphenols from star anise extracts.

The developed HPLC method using binary gradient solvent systems (acetonotrile:methanol, 70:30 and glacial acetic acid:water, 0.5:99.5) and C_18_ column (250 × 4.6 mm internal diameter, 5 µm particle size) could simultaneously separate thirteen phenolic acids and five flavonoids from SAT within 25 min at a flow rate of 0.8mL/min (Figure 1). The concentration (mg/100 mL of tea) of identified phenolic acids and flavonoids in SAT is presented in Table 2. *p*-Coumeric acid (74.46 mg/100 mL of tea) was the major phenolic acid in the SAT followed by gallic aid (35.24 mg/100 mL of tea), cinamic acid (10.85 mg/100 mL of tea), chlorogenic acid (8.79 mg/100 mL of tea) and ferulic acid (6.15 mg/100 mL of tea). Besides these major phenolic acids, salicylic acid (3.69 mg/100 mL of tea), 4-hydroxybenzoid acid (2.99 mg/100 mL of tea), caffeic acid (1.87 mg/100 mL of tea), benzoic acid (0.97 mg/100 mL of tea), sinapic acid (0.93 mg/100 mL of tea), ellagic acid (0.91 mg/100 mL of tea) and syringic acid (0.63 mg/100 mL of tea) were also identified in the SAT. Moreover, Catechin (85.51 mg/100 mL of tea) and rutin (67.91 mg/100 mL of tea) were the major flavonoids detected in the SAT along with quercetin, kaempferol and myricetin (Table 2).

Our results are also in agreement with the finding of Aly et al. [20], who reported rutin (1112.6 mg/100 g DW) was the major identified phenolic compound in star anise, followed by ferulic acid (103.23 mg/100g DW), catechin (75.64), gallic acid (62.89), caffeic acid (46.42), and cinnamic acid (16.90 mg/100g DW).

### 3.3. Effect of SAT on a High-Fat Diet-Induced Obesity Model

#### 3.3.1. Effect on Body Weight, Organs Weights and Their Indexes

The initial and final body weight, the percent increase in body weight of different rat groups and body mass index (BMI) are presented in Table 3. This high-fat diet group presented 99.30% increase in body weight while a 40.64% increase in the normal control (NC) group. The BMI of the HFDC group was significantly greater (*p* ≤ 0.05) than NC group (0.60 g/cm^2^). High-fat diet control (HFDC) group showed a significant (*p* ≤ 0.05) increase in body weight and BMI, validating the current study’s adopted model of obesity by using high-fat diet. Oral administration of orlistat drug and SAT significantly (*p* ≤ 0.05) slowdown the augmentation in both the body weight and the BMI in all treatment groups. Orlistat group (PC) showed the maximum effect on the increase in body weight (57%) and BMI (0.57 g/cm^2^) whereas higher doses of SAT showed better effects on the increase in body weight (75%) and BMI (0.64 g/cm^2^) as compared to lower doses of SAT. Table 3 shows that the weight of the body organs was also affected by the high-fat diet. The HFDC group demonstrated a significantly increased (*p* ≤ 0.05) in the kidney and liver tissues’ total weight when compared to that of the NC group. The higher doses of SAT and orlistat reduced the organ weights and kidney and liver indexes but the effect was not significant (*p* > 0.05). 

Although weight gain and obesity is a complex process, the consumption of high-fat and high calorific diets is one of the major factors that develop obesity [9]. The consumption of a high-fat diet may increase the storage of triglycerides in adipose tissue, leading to an increase in fat mass and body weight [36]. According to Velez-Carrasco et al. [37], a reduction in body weight of about 5–10% can have a significant effect on health status. The increase in body weight in the HFSD group is believed to be due to a high-calorie diet (saturated fats) and drinking water, which resulted in the deposition of body fat pads.

#### 3.3.2. Effect on Serum Lipid Profile

**Figure 2:**
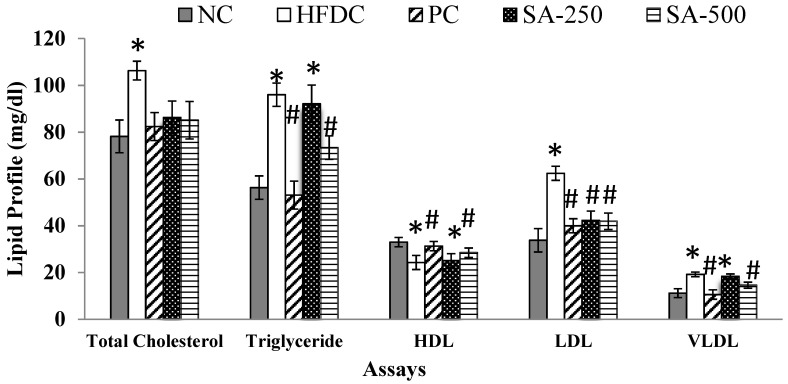
Effect of different treatments on the lipid metabolic profile on different rat groups. * *p* ≤ 0.05 difference compared to NC and ^#^ *p* ≤ 0.05 when compared to HFDC group.

The metabolic profile was assessed by measuring the total cholesterol (TC), triglycerides (TG), HDL, LDL and VLDL were measured in the plasma of all the treatment and control groups and presented in Figure 2. As shown in the figure, the HFDC group had significantly (*p* ≤ 0.05) increased the serum levels of TC (106.3 mg/dL), TG (96.0 mg/dL), LDL (62.4 mg/dL) and VLDL (19.2 mg/dL) and had decreased levels of HDL (24.3 mg/dL), when compared with the NC showing the effectiveness of the model. Serum levels of TC, TG, LDL, VLDL and HDL in NC rats were 78.2, 56.3, 33.8, 11.2 and 33.0 mg/dL, respectively.

All the treatment groups significantly (*p* ≤ 0.05) reduced the elevated levels of serum TC. Treatment group PC and SA-500 showed significant (*p* ≤ 0.05) reduction in the TG, LDL and VLDL levels and recovered HDL level when compared to the HFDC group (Figure 2). A protective effect was shown by SAT (500 mg/kg BW) vs. PC groups in the plasma where TC, TG, HDL, LDL and VLDL levels were 85.1 vs. 82, 73.4 vs.53, 28.5 vs. 31, 41.9 vs. 40 and 14.6 vs. 11 mg/dl respectively as shown (Figure 2). Furthermore, the serum total cholesterol concentrations in all treated groups were not significantly different from the normal control group at the end of the treatment.

Increased lipid profile levels in the high-fat diet could be because of the activation of gastric lipases enzyme, which further leads to increased fat absorption in the intestine While elevated TG levels can be speculated due to reduce fatty acid oxidation leading to increase levels of hepatic triglycerides [17]. Elevated levels of HDL cholesterol are beneficial in the cholesterol excretion from the liver in the bile whereas, elevated levels of bad cholesterol (LDL) and TC increase the potential risk of coronary heart diseases (CAD) [17]. One of the possibilities of increased LDL-cholesterol may be due to the reduced expression of the LDL-receptor sites in response to a high-fat diet [38]. Therefore, lowering the LDL cholesterol levels may be an important factor in lowering the serum total cholesterol level in high-fat diet-fed rats. There is no earlier report available on the lipid profile levels of rats fed with the aqueous extract of star anise to compare the results of our present analysis. The antihyperlipidemic effect of SAT may be related to the presence of high polyphenol concentration that is able to stimulate thermogenesis and decrease fat accumulation which may perhaps be related to its ability to inhibit pancreatic lipase activity [36]. The reduction of LDL cholesterols in SAT could be due to reducing the suppressive activity of the high-fat diet on the LDL-receptor site. 

#### 3.3.3. Effect on Oxidative Stress Parameters

The effect of SAT on oxidative stress system parameters like MDA, SOD, GSH, and TAC levels of all the treatments and control groups were accessed and shown in Table 4. The plasma levels of MDA in the rats of the high-fat and sugar-diet control (HFSDC) group were significantly higher (*p* ≤ 0.05) when compared to the same with the rats of the NC group, thus showing the elevation of oxidative stress in tested animals. All the treatment groups including the PC group showed a significant (*p* ≤ 0.05) decrease in the elevated levels of MDA. Daily administration of SAT effectively controlled the generation of MDA in rat groups and the best effect was shown in the SA-500 group, which was comparable to the PC (orlistat) group (3.2 nmol/L). The serum SOD level in the HFSDC group was found to be 120.1 U/mL, which is significantly lesser (*p* ≤ 0.05) than the NC group (159.0 U/mL). SA-500 and PC groups showed protective effects, and the levels of SOD were found to be 133.3 and 137.1 U/mL, respectively. A significant reduction (*p* ≤ 0.05) in the level of GSH was recorded in the HFSDC group (123.7 mg/L) as compared to the NC group (160.1 mg/L) which leads to oxidative stress. SA-500 and PC (orlistat) groups showed a protective effect against oxidative stress and the level of GSH in the SA-500 group was comparable with PC (149.2 mg/L). Total antioxidant capacity was significantly decreased (*p* ≤ 0.05) in the HFSDC group when compared to the NC group (1.42 mmol/L vs. 1.90 mmol/L). Treatment groups SA-500 and PC showed a significant (*p* ≤ 0.05) increase in the TAC values (Table 4). SA-500 showed 1.63 mmol/L TAC that is comparable with PC (1.70 mmol/L).

Lipid peroxidation is an important biomarker to access oxidative stress and the determination of MDA, SOD GSH levels and reflects the degree of lipid peroxidation/oxidative stress [39]. The reduction in the plasma levels of MDA in the HFSDC group may be due to lipid peroxidation and the reduced plasma levels of the MDA in the treatment groups show the antioxidant potential of SAT, which controls lipid peroxidation. Superoxide dismutase (SOD) is a metalloenzyme that catalyzes the dismutation of superoxide radicals and decreases the levels of SOD showing signs of oxidative stress [40]. GSH is a natural antioxidant present in the body and acts against oxidative stress due to free radicals and peroxides [39]. The oxidative stress and obesity are indicated by the marked reduction in GSH content due to the impairment of H_2_O_2_ clearance and promotion of hydroxyl radical (•OH) formation [39]. Levels of SOD and GSH were significantly reduced (*p* < 0.05) in the HFSDC group as compared to the normal control group showed the stress level in the group [39,40]. SA-500 and PC groups restored the reduced plasma levels of SOD and GSH thus showing potential against oxidative stress. Clinically, TAC, total antioxidant capacity, has been widely used to assess oxidative stress and serum antioxidant depletion [41]. Decreased plasma levels of TAC in HFSDC group rats may be because of either increased oxidative stress or decreased availability of antioxidants. This imbalance was restored with the administration of SAT through decreasing free radical generation and increasing antioxidant levels. Several studies reported that polyphenol-rich food and drinks provide the strength to combat oxidative stress through various modes of action including microbiota modulation [42].

#### 3.3.4. Effect on Serum Levels of Liver and Kidney Enzymes

The effect of SAT on the biochemical parameters of different rat groups is presented in Table 5. Serum levels of aspartate aminotransferase (AST) were increased (98.9 µ/L) while the level of total bilirubin (BT) was decreased (0.28 mg/dL) significantly (*p* ≤ 0.05) in the rats of HFDC group, when compared with the NC group, that is, 63.3 µ/L and 0.45 mg/dl, respectively (Table 5). However, the increase in the level of alanine aminotransferase (ALT) was not significant (*p* > 0.05). Both SAT groups decreased the elevated levels of ALT and AST and increased the level of BT, thus showing the protective effect that is comparable with the PC (orlistat) group. The AST (µ/L), ALT (µ/L), BT (mg/dL) levels of SA-500, SA-250 and PC groups were 74.4, 88.4, 63.0; 71.4, 77.1, 62.5; 0.34, 0.31, 0.38, respectively. The major effect appeared in the SA-500 group that is comparable with the PC group. Alkaline phosphate (AP) and serum creatinine (SC) levels were significantly increased (*p* ≤ 0.05) in the HFDC group (164 µ/L and 0.56 mg/dL, respectively) when compared to the control group (Table 5). Both the SAT doses showed a protective effect and decreased the elevated level of AP and SC. SA-500 group showed the best effects, for example, AP 153 µ/L and SC 0.41 mg/dL, which was comparable with the PC group (Table 5). 

In cases of obesity where oxidative stress is increased, consumption of bilirubin increases, leading to a reduction in BT level in serum which results in an increased risk of cardiovascular diseases by causing endothelial dysfunction [43]. Both SAT doses and orlistat drug reduced the high serum levels of ALT and AST and increased the bilirubin level. Previously published data [44] reported the negative association of serum bilirubin levels with abdominal obesity. Chang et al. [45] reported that serum direct bilirubin levels were inversely associated with low-density lipoprotein, total cholesterol, and triglyceride and positively associated with HDL.

### 3.4. Histopathological Evidence

**Figure 3:**
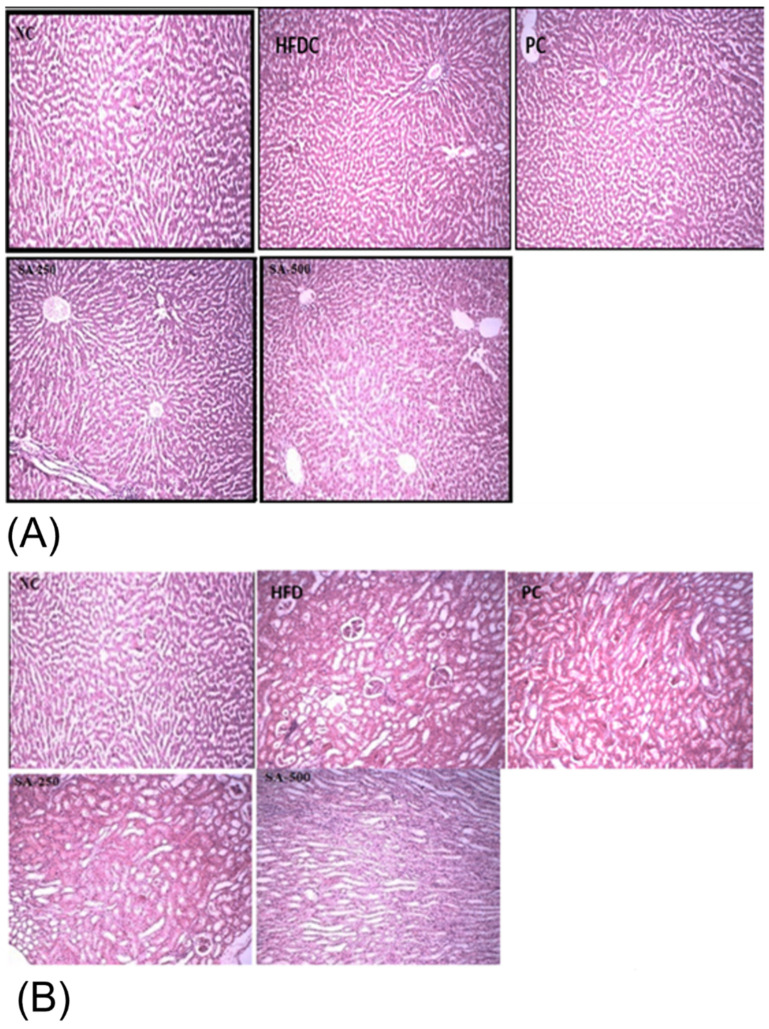
Histopathological images of (**A**) liver and (**B**) kidney of different rat groups showing the morphological changes. NC, normal control; HFDC, high fat diet control; PC, positive control; SA-250 and SA-500, Star anise 250 and 500 mg/kg BW.

Acute study showed that rats of all the groups were active, healthy, with no signs of loss of hair, no redness of the eyes, no moribund and hunched back signs after oral administration of SAT. Histopathological evidence showed that the liver of all the treatment groups showed no ballooning, nuclei were of normal shape, and there were no inflammatory cells (Figure 3A). Similarly, the kidneys of all the groups showed normal tubules, glomerulus, and parenchyma as shown in Figure 3B. Histopathological evaluation of biopsy specimens is an authentic diagnostics tool for the fatty liver and hepatocellular injury that is diagnosed by hepatocellular ballooning and illustrated as swollen hepatocytes with rarefied cytoplasm [46].

## 4. Conclusions

The present study demonstrated that the use of SAT at doses 250 and 500 mg/kg showed an anti-obesity effect that was comparable with the standard anti-obesity drug (orlistat), especially when a high dose (500 mg/kg) was used. Based on these results, it can be concluded that the selected SAT may have therapeutic potential to be used as an anti-obesity agent because of the ease of availability and perceived lesser side effects than synthetic anti-obesity drugs. The possibility of using these herbal infusions as supplementation with current anti-obesity drugs can reduce the dosage to overcome the possible side effects of these drugs.

## Figures and Tables

**Table 1:** Aqueous extract yield, total phenolic content and total flavonoid contents and DPPH radical scavenging activity of star anise tea.

Assays	Star Anise	BHT
Extract Yield (g/100g)	4.83 ± 0.28	----
TP (mg/g)	0.83 ± 0.05	----
TF (mg/g)	1.24 ± 0.08	----
DPPH radical scavenging activity (%)	51.3 ± 2.3 ^a^	89 ± 4 ^c^

The values are mean ± SD (n = 3). Different alphabets in superscript in the same row represent significant (*p* ≤ 0.05) differences among star anise tea and BHT.

**Table 2:** Contents of phenolic acids and flavonoids identified from star anise tea by Rp-HPLC.

Compounds	Concentration of Compounds (mg/100 mL of Tea)
Gallic acid	35.24 ± 1.67
4-Hydroxybenzoic acid	2.99 ± 0.15
Catechin	85.51 ± 3.45
Chlorogenic acid	8.79 ± 0.40
Caffeic acid	1.87 ± 0.09
Syringic acid	0.63 ± 0.03
Vanillic acid	8.58 ± 0.45
*p*-Coumaric acid	74.16 ± 2.36
Salicylic acid	3.69 ± 0.19
Rutin	67.91 ± 2.23
Sinapic acid	0.93 ± 0.07
Ferulic acid	6.15 ± 0.24
Ellagic acid	0.91 ± 0.05
Cinamic acid	10.85 ± 0.42
Benzoic acid	0.97 ± 0.05
Myricetin	0.41 ± 0.02
Quercetin	5.52 ± 0.13
Kaempferol	2.44 ± 0.13

The values are mean ± S.D of three independent experiments.

**Table 3:** Effect of herbal tea and orlistat on the body, kidney and liver weights, body mass, kidney and liver index of different groups of obesity rat model.

Groups	Body Weight			BMI (g/cm^2^)	Kidney Weight (g)	Kidney Index (%)	Liver Weight (g)	Liver Index (%)
	Initial (g)	Final (g)	Increase (%)					
NC	155 ± 15 ^a^_a_	218 ± 12 ^a^_b_	41	0.60 ± 0 ^b^	1.6 ± 0.2 ^a^	0.71	7 ± 1 ^a^	3.17
HFDC	143 ± 14 ^a^_a_	285 ± 12 ^b^_b_	99	0.79 ± 0.0 ^d^	1.7 ± 0.2 ^a^	0.61	10 ± 1 ^b^	3.54
PC	145 ± 11 ^a^_a_	228 ± 21 ^a^_b_	57	0.57 ± 0.0 ^ab^	1.6 ± 0.2 ^a^	0.68	8 ± 1 ^ab^	3.45
SA-250	147 ± 12 ^a^_a_	270 ± 11 ^b^_b_	84	0.68 ± 0.0 ^c^	1.7 ± 0.2 ^a^	0.61	9 ± 1 ^ab^	3.49
SA-500	147 ± 11 ^a^_a_	257 ± 11 ^ab^_b_	75	0.64 ± 0.0 ^bc^	1.7 ± 0.2 ^a^	0.64	8 ± 1 ^ab^	3.41

The values are mean ± SD (n = 6). Different alphabets in superscript in the same column show significant (*p* ≤ 0.05) difference among all the treatment and control groups. Different letters in subscript in the initial and final body weight of rats (row-wise) show significant (*p* ≤ 0.05) difference among all the treatment and control groups. NC, normal control; HFDC, high fat diet control; PC, positive control; SA-250 and SA-500, Star anise 250 and 500 mg/kg BW, BMI, Body Mass Index.

**Table 4:** Effect of star anise tea and orlistat treatment on the oxidative stress parameters of different groups of obesity rat model.

Groups	Oxidative Stress Parameters			
	MDA (nmol/L)	SOD (U/mL)	GSH (mg/L)	TAC (mmol/L)
NC	2.7 ± 0.2	159.0 ± 8.2	160.1 ± 11	1.90 ± 0.2
HFDC	7.0 ± 0.4 *	120.1 ± 9.3 *	123.7 ± 10 *	1.42 ± 0.1 *
PC	3.2 ± 0.2 *^#^	137.1 ± 8.0 *	149.2 ± 9 *^#^	1.70 ± 0.2 ^#^
SA-250	4.8 ± 0.2 *^#^	129.0 ± 10.1 *	139.0 ± 7 *	1.50 ± 0.1
SA-500	3.9 ± 0.3 *^#^	133.3 ± 10.4 *	145.0 ± 9 ^#^	1.63 ± 0.1 *^#^

* shows significant (*p* ≤ 0.05) difference compared to NC and ^#^ show significant (*p* ≤ 0.05) difference compared to HFSDC groups. NC, normal control; HFSDC, high fat and sugar diet control; PC, positive control; SA-250 and SA-500, Star anise 250 and 500 mg/kg BW; MDA, malondialdehyde; SOD, superoxide dismutase; GSH, reduced glutathione; TAC, total antioxidant capacity.

**Table 5:** Effect of star anise tea and orlistat treatment on the biochemical parameters of different groups of obesity rat model.

Groups	Liver Parameters			Kidney Parameters	
	AST (µ/L)	ALT) (µ/L)	BT (mg/dL)	AP (µ/L)	SC (mg/dL)
NC	63.3 ± 5	81.7 ± 4	0.45 ± 0.05	143 ± 10	0.41 ± 0.06
HFDC	98.9 ± 5 *	82.3 ± 5	0.28 ± 0.02 *	164 ± 9 *	0.56 ± 0.04 *
PC	63.0 ± 6 ^#^	62.5 ± 4 *^#^	0.38 ± 0.03 ^#^	152 ±11	0.42 ± 0.06 ^#^
SA-250	88.4 ± 4 *^#^	77.1 ± 3	0.31 ± 0.03 *	158 ± 15	0.44 ± 0.05 ^#^
SA-500	74.4 ± 5 *^#^	71.4 ± 4 *^#^	0.34 ± 0.03 *^#^	153 ± 15	0.41 ± 0.02 ^#^

The values are mean ± standard deviation of six rats of same group. * Showing significant (*p* ≤ 0.05) difference compared to NC and ^#^ show significant (*p* ≤ 0.05) difference compared to HFSDC groups. NC, normal control; HFDC, high fat diet control; PC, positive control; SA-250 and SA-500, Star anise 250 and 500 mg/kg BW; BT, Bilirubin total; ALT, Alanine aminotransferase; APT, Aspartate aminotransferase; SC, Serum cretanine; AP, Alkaline phosphatase.

## Data Availability

Not applicable.
